# Eosinophils in glioblastoma biology

**DOI:** 10.1186/1742-2094-9-11

**Published:** 2012-01-17

**Authors:** Colleen S Curran, Paul J Bertics

**Affiliations:** 1 Department of Biomolecular Chemistry, University of Wisconsin School of Medicine and Public Health, Madison, WI 53706, USA

**Keywords:** eosinophil, glioblastoma, RAGE, S100 proteins, atopy, allergy, asthma

## Abstract

Glioblastoma multiforme (GBM) is the most common primary brain tumor in adults. The development of this malignant glial lesion involves a multi-faceted process that results in a loss of genetic or epigenetic gene control, un-regulated cell growth, and immune tolerance. Of interest, atopic diseases are characterized by a lack of immune tolerance and are inversely associated with glioma risk. One cell type that is an established effector cell in the pathobiology of atopic disease is the eosinophil. In response to various stimuli, the eosinophil is able to produce cytotoxic granules, neuromediators, and pro-inflammatory cytokines as well as pro-fibrotic and angiogenic factors involved in pathogen clearance and tissue remodeling and repair. These various biological properties reveal that the eosinophil is a key immunoregulatory cell capable of influencing the activity of both innate and adaptive immune responses. Of central importance to this report is the observation that eosinophil migration to the brain occurs in response to traumatic brain injury and following certain immunotherapeutic treatments for GBM. Although eosinophils have been identified in various central nervous system pathologies, and are known to operate in wound/repair and tumorstatic models, the potential roles of eosinophils in GBM development and the tumor immunological response are only beginning to be recognized and are therefore the subject of the present review.

## Introduction

### Glioblastoma (GBM), atopy, and the immune response

Cancer is a multi-faceted cellular process involving the exploitation of genetic and/or epigenetic DNA modifications by a microenvironment that endows immune privilege. This process has been characterized as occurring in three stages known as initiation, promotion and progression where each stage respectively relates to the biological events required for the formation, proliferation and migration of altered immortal cells [1]. Each of these developmental stages may be influenced by immune cells [2]. Although the brain has historically been considered an organ of immune privilege [3], recent research indicates that immune cells may play a pivotal role in both the development of and host defense against brain tumors [4-6].

In U.S. adults, primary brain tumors account for 2% of all cancers, yielding approximately 22,000 diagnoses and 13,000 deaths annually [7]. The developmental pathologies of brain tumors are diverse and may be influenced by age, gender, environmental factors and/or genetic predispositions. These tumors may be classified as glioma (astrocytoma, oligodendroglioma, ependymomas) or non-glioma (meningiomas, pituitary tumors and medulloblastomas) [8]. Gliomas account for approximately 30% of all brain tumors and 80% of malignant brain tumors [9]. Glioblastoma multiforme (GBM) is the most common malignant glioma, and is generally lethal within one year after diagnosis [10].

**Figure 1:**
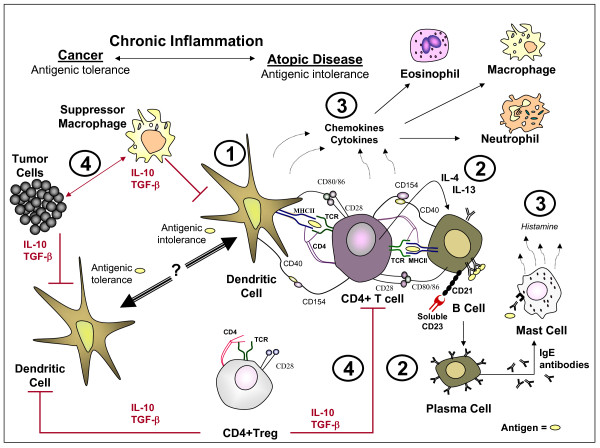
**The immune response in cancer and atopic disease**. **(1) **Full activation of antigen presentation cells (APCs: e.g. Dendritic cell, B cell) and T cells. **(2) **T cell cytokines (IL-4, IL-13) and soluble CD23 ligation to CD21, induces B cell differentiation, the generation of plasma cells, the production of IgE, and the subsequent IgE-dependent activation of mast cells. **(3) **Activated mast cells, APCs, and T cells produce chemokines and cytokines that recruit granulocytic cells (eosinophils, macrophages, neutrophils). **(4) **Immuno-suppressive cytokines (IL-10, TGF-β) are produced by tumor cells, suppressor macrophages, and T regulatory (CD4+ Treg) cells. These cytokines and additional mediators or cell:cell interactions prevent a specific adaptive immune response required in tumor eradication (see text for additional details).

Treatment of GBM is confounded by the complex nature of the tumor and the tumor microenvironment. GBM tumor cells have been indicated to evade surgical, radiotherapeutic, chemotherapeutic and immunotherapeutic interventions by respectively infiltrating into the surrounding brain tissue, down-regulating tumor suppressor proteins, up-regulating DNA repair enzymes, and producing immunosuppressive cytokines [11]. Tumor evasiveness is also thought to involve chronic inflammation and the recruitment of myeloid suppressor cells and T-regulatory cells that effectively obstruct innate and adaptive anti-tumor immune responses [6,12]. This antigenic tolerance is lacking in atopic diseases [13] which reportedly have an inverse association with glioma risk [14-23] (see Table 1). In the atopic immune response, aberrant recognition of antigen/allergen by antigen presentation cells (APCs: e.g. Dendritic cell, B cell) allows for the processing of antigen/allergen into a peptide for presentation via major histocompatibility complex (MHC) [24]. Full activation of APCs requires MHC plus peptide interaction with the T cell receptor (TCR) and CD40 ligation whereas full activation of T cells requires MHC:peptide:TCR interaction and CD28 ligation to CD80/86 [25]. Further interactions involving the B cell surface marker, CD21, with soluble CD23 and B cell cytokine stimulation (IL-4, IL-13) induces the generation of plasma cells specific for the immunoglobulin IgE, the most common immunoglobulin in allergy and asthma [26,27]. High serum CD23 and IgE levels are associated with increased GBM patient survival [28,29]. These molecules are also found to activate of mast cells and eosinophils [30]. Cytokines and chemokines produced by activated mast cells, T cells, and APCs increase vascular permeability for the enhanced recruitment of granulocytic immune cells (e.g., eosinophils, macrophages, and neutrophils) and the development of chronic inflammation [31]. In the tumor immunological response, chronic inflammation is also found to occur but the biological features distinctly differ [12]. Immuno-suppressive cytokines (IL-10, TGF-β) secreted by tumor cells, suppressor macrophages, and T regulatory (CD4+ Treg) cells that in association with additional mediators or cell:cell interactions inhibit the pro-inflammatory functions of dendritic cells, provoke chronic inflammation associated with tumorigenesis, and prevent a specific adaptive immune response required in tumor eradication [12,32]. Thus, the distinct immune activation parameters in an allergic response may be imperative to immunotherapeutic treatments in cancer. In this regard, a comparison of the immune responses observed in cancer versus atopic diseases is summarized in Figure 1.

**Table 1:** Case studies assessing the association between atopic disease and glioma

Number of glioma cases	Relationship between atopic disease and glioma risk **Relative risk (RR), Odds ratio (OR)**, Confidence interval (CI)	Year/ Reference
1,178	Allergy: RR = 0.59, 95% CI: 0.49-0.71 Asthma: RR = 0.75, 95% CI: 0.55-1.03 Eczema: RR = 0.64, 95% CI: 0.47-0.86	1999/[14]

405	Allergy: OR = 0.47, 95% CI: 0.33-0.67	2002/[15]

489	Allergy: OR = 0.67, 95% CI: 0.52-0.86 Asthma: OR = 0.63, 95% CI: 0.43-0.92 Eczema: OR = 0.76, 95% CI: 0.45-1.27	2002/[16]

965	Allergy: OR = 0.65, 95% CI: 0.47-0.90 Asthma: OR = 0.71, 95% CI: 0.54-0.92 Eczema: OR = 0.74, 95% CI: 0.56-0.97	2006/[17]

1,527	Allergy: OR = 0.70, 95% CI: 0.61-0.80 Asthma: OR = 0.65, 95% CI: 0.51-0.82 Eczema: OR = 0.65, 95% CI: 0.54-0.79	2007/[18]

3450	Allergy: OR = 0.61, 95% CI: 0.55-0.67 Asthma: OR = 0.68, 95% CI: 0.58-0.80 Eczema: OR = 0.69, 95% CI: 0.58-0.82	2007/[19]

535	Allergy: OR = 0.59, 95% CI: 0.41-0.85	2009/[20]

366	Allergy: OR = 0.92, 95% CI: 0.70-1.22 Asthma: OR = 0.65, 95% CI: 0.36-1.19 Eczema: OR = 0.91, 95% CI: 0.65-1.27	2009/[21]

388	Allergy: OR = 0.34, 95% CI: 0.23-0.50 Asthma: OR = 0.96, 95% CI: 0.58-1.59 Eczema: OR = 0.70, 95% CI: 0.30-1.64	2009/[22]

855	Allergy: OR = 0.62, 95% CI: 0.51-0.76	2011/[23]

### Eosinophils

Eosinophils are myeloid cells known to accumulate at specific sites, such as the lung and gastrointestinal tract, in the pathobiology of atopic disease [33]. The functions of eosinophils are diverse and include organ development, tissue homeostasis, antigen presentation, wound repair, tissue remodeling, cytotoxic clearance of pathogens, nerve growth, and the production of various chemokines and cytokines known to influence both innate and adaptive immune responses [34-36]. In asthma, eosinophil recruitment has been characterized by early phase IgE-mediated activation of mast cells, the production of pro-inflammatory cytokines (e.g.: IL-2, IL-4, IL-5, GM-CSF) and the late phase recruitment of Th2 cells and eosinophils [37]. These events are preceded by the generation of IgE producing plasma cells (see Figure 1). Murine *in vitro *and *in vivo *studies suggest that eosinophils are required for the long-term maintenance of plasma cells [38,39]. Cytokines produced by mast cells or CD4+ T cells (e.g.: IL-3, IL-5, GM-CSF) are known to induce the differentiation, activation, and survival of eosinophils [40,41]. Activated eosinophils produce cytotoxic mediators, pro-inflammatory cytokines, pro-fibrotic and angiogenic factors that may alter innate (basophils, mast cells, neutrophils, dendritic cells) and adaptive (T cells) immune responses [35,42]. The activation state of eosinophils may therefore also affect the tumor microenviroment and tumor development.

### Eosinophils and cancer

Previous research has suggested that organs interfacing with the external environment (i.e., mouth, gastrointestinal tract, cervix) are more likely to exhibit inverse associations between allergy and cancer risk than non-interfacing organs (i.e., ovary, breast, prostate) [43]. The possible interaction of neural stem cells and glial progenitor cells with airborne pathogens via the olfactory bulb in the lateral ventricles of the brain suggests that glioma development may also be precluded as a result of an interface with the external environment [43,44]. Eosinophils are an established effector cell in atopic disease [33] and may therefore participate in the reported inverse associations between atopic disease (allergy, asthma, eczema) and the risk of glioma (see Table 1), oral cancers [45], and gastrointestinal tract cancers [46,47]. Despite differences in clinical protocols, organ microenvironment, and measurements to identify eosinophilia, a link between certain tumors (colon, stomach, brain, oral/mouth, penile, and uterine/cervix; see Table 2) and eosinophilia has been identified at various stages of disease progression and in association with enhanced patient survival [48-57]. Similar studies with respect to genitourinary cancers, however, are not uniform in outcome [43] (see Table 2). This observation may be a result of patient exposure to certain viruses [58], the organ interface with the external environment [43], hormonal influence [59,60] or a lack of relevant investigational research. Of interest, eosinophilia in human cancers involving the immune system (Hodgkin's disease, cutaneous T cell lymphoma (CTCL)) has been associated with reduced patient survival (Table 2). The progression of these same diseases is positively influenced by immune factors (IgE, IL-5) known to promote the allergic response and induce the recruitment and activation of eosinophils [30,37,49,61-63]. These human cancers also exhibit a strong Th2 (CD4+) response but lack specific Th1 cytotoxic T cell (CD8+) populations [64,65]. Human atopic diseases are characterized not only by CD4+ T cell influx but also by CD8+ T cell effector functions [66,67], suggesting that the eosinophilia associated with adaptive CD8+ T cell immune responses may be essential in the host defense to tumors and allergens.

**Table 2:** Identification of eosinophilia in human cancers

Cancer Type	Treatment	Eosinophil localization	Outcome
Colonic epithelial neoplasms [48]	Resection	Tumor tissue	Tissue eosinophilia significantly identified in adenomas was not found in invasive carcinomas.

Cutaneous T Cell Lymphoma (CTCL)[49]	Physical exam and blood draw	Blood	Patients in the late stages of CTCL were found to have significantly elevated IgE levels and eosinophilia.

Gastric cancer [50]	Gastrectomy with lymph node dissection without preoperative irradiation and immunochemotherapy	Blood, tumor tissue	Tissue eosinophilia was significantly associated with poorly differentiated tumors and increased patient survival. The degree of eosinophilic infiltration into tumors correlated with blood eosinophilia.

Hodgkin's disease [51]	Chemotherapy and/or radiation	Diagnostic lymph nodes	Clinical outcome was significantly worse for patients with tissue eosinophilia

Malignant glioma [52]	IL-2 combined with ex vivo activated autologous killer cells was infused via an indwelling catheter placed into the surgical resection cavity.	Intracavitary fluid, inracavitary tissue, cerebral spinal fluid	Immunotherapy induced eosinophilia in the intracavitary fluid, tissue, and cerebral spinal fluid. Identified eosinophilia appeared to correlate with longer patient survival.

Non-hematological cancers that had either failed conventional therapy or for which no standard therapy exists [53]	Simultaneous subcutaneous injections of IL-2 and IL-4 were given 5 days a week for 3 consecutive weeks followed by a 1 week rest period = 1 cycle.	Blood samples were drawn before the start of therapy and at the completion of each cycle of treatment.	Eosinophilia of unknown significance occurred in all patients and was generally highest when measured on the fifth day of the third treatment week.

Oral squamous cell carcinoma [54]	Resection	Tumor tissue of the oral tongue, floor of the mouth, retromolar area and inferior gingiva	Tissue eosinophilia may represent a favorable prognostic factor in clinical stage II and III oral squamous cell carcinomas from the floor of the mouth, oral tongue, retromolar area, and inferior gingiva.

Penile cancer [55]	Partial penectomy, circumcision, lymphadenectomy and/or irradiation depending upon staging	Tumor tissue	Penile cancer patients with tissue eosinophilia tended to live longer. Eosinophils were identified at a higher rate in stages I and II than in stages III and IV.

Renal cell carcinoma [56]	IL-2 was given subcutaneously for 5 days per week, together with interferon-alpha by intramuscular route twice weekly, for 4 consecutive weeks corresponding to one treatment cycle.	Blood	Pre-treatment and post-treatment eosinophilia was a predictive indicator of immunotherapy failure.

Uterine cervix carcinoma [57]	Hysterectomy	Tumor tissue	Eosinophilia was associated with statistically improved survival in women with stage IB cervical carcinomas.

### Eosinophils and GBM

**Figure 2:**
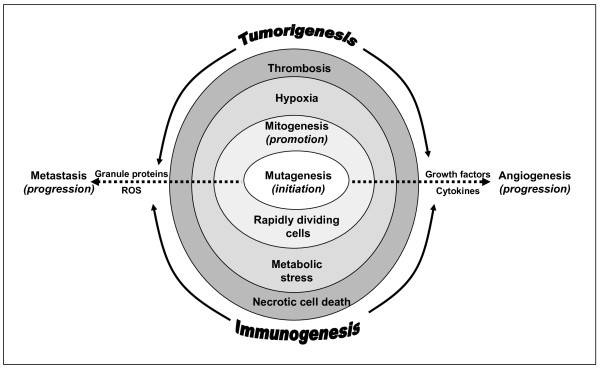
**The potential role(s) of tumor associated eosinophils**. Tumor development has been characterized as proceeding through several stages (initiation, promotion, progression). The initiation stage is a period of mutagenesis where genetic and/or epigenetic alternations in stem cells or progenitor cells are established. The promotion stage invokes cellular growth (mitogenesis) that is induced by growth factors and altered apoptotic cell signal pathways. This increased cell division creates a microenvironment of metabolic stress, hypoxia and necrotic cell death that has also been associated with thrombosis. The progression stage involves additional genetic and epigenetic events that confer phenotypic changes necessary for tumor cell autonomous growth, invasiveness, and migration. Eosinophils are able to produce growth factors, cytokines, chemokines, blood coagulants, and cytotoxic mediators that may affect each stage of tumor development.

Eosinophils accumulate in various human central nervous system disorders (eosinophilic meningoencephalitis, idiopathic hypereosinophilic syndrome encephalopathy, eosinophilic meningitis, peripheral neuropathy), including tumors of the brain (neuroblastoma, leiomyoma, glioblastoma) [52,68-75]. Interestingly, eosinophilic meningitis has been identified in a case of disseminated GBM [74]. Eosinophils have also been shown in an *in vivo *murine model to be recruited to necrotic tissue [76], which is also a primary determinant of human GBM [77]. Clinico-pathological assessment of human eosinophil migration to the brain has been indicated to occur in the development of subdural hematomas [78,79], a condition that emerges in response to increased intracranial pressure in some human GBM case studies [80,81]. Marked eosinophilia in sediments of spinal fluid has been identified in patients with intracerebral neoplasms, including one case of GBM [82]. In two separate clinical trials, enhanced GBM patient survival was associated with tissue eosinophilia found after postoperative treatments with interleukin-2 (IL-2) [52,75]. Human eosinophils in an *in vitro *study have also been reported to be responsive to S100B [83], a possible blood marker in some GBM cases that is known to be released by CD8+ T cells, astrocytes, oligodendrocytes, and tumor cells [84-86]. However, the mechanisms by which eosinophils may function in tissue destruction or remodeling and repair are not clearly understood [35,87]. Thus, the purpose of this review is to examine the potential roles of eosinophils in the stages of GBM development and the tumor immune response (see Figure 2).

## Initiation

### Known factors in initiating GBM tumor formation

Malignant gliomas are thought to originate from neuroectodermal stem cells or tumor progenitor cells [88]. Genetic and/or epigenetic alterations in these cells promote the dysregulation of several signaling molecules/networks involving intracellular (MDM2, PTEN, TP53, annexin A7) and extracellular (platelet-derived growth factor, epidermal growth factor, vascular endothelial growth factor, fibroblast growth factor) protein function [88,89]. While the development of this genomic instability is not clearly known, rare genetic disorders (Li-Fraumeni syndrome, neurofibromatosis, Turcot's syndrome), ionizing radiation, and oxidative stress from toxic chemical exposure or biological aging have been implicated in gliomagenesis [90-93]. Evidence supporting human cytomegalovirus (HCMV) infections and interleukin-4 receptor alpha (IL-4Rα) or IL-13 single nucleotide polymorphisms (SNPs) as GBM risk factors have also been found [94-98], suggesting that inflammation may play a role in GBM etiology.

### IL-4 and cancer

The cytokines, IL-4 and IL-13, share a common receptor component, the IL-4Rα, and can initiate many similar immune responses [99]. These cytokines also play a pivotal role in the allergic response by stimulating B cell IgE synthesis and inducing epithelial production of pro-eosinophilic chemokines (eotaxin, monocyte chemotactic protein-1 (MCP-1)) [99]. As a result, clinical assessment of anti-IL-13 antibodies is being explored in the treatment of asthma with some improvement in lung function indicated [100]. However, in one asthmatic study involving 56 patients, the recruitment of eosinophils was not affected by anti-IL-13 therapy [101], perhaps due to alternative responses via IL-4 [99]. In a Phase I clinical trial of IL-4 involving cancer patients with non-hematological refractory malignancies, systemic eosinophil degranulation was identified in patient serum, urine, and skin biopsies of rashes via increased identification of the eosinophil granule protein, major basic protein (MBP) [102]. Patient sera were also examined for eosinophil viability factors where the cytokines IL-5, IL-3 and GM-CSF were identified in mediating the response [102]. Because of the identified activity of eosinophils, additional Phase I and II trials were explored but no significant tumor response was obtained in examinations of refractory malignancies [103,104]. Further assessment involving the co-administration of IL-4 and IL-2 resulted in eosinophilia of unknown significance in all patients studied [53] (see Table 2). Efforts to understand eosinophilia and the tumor response to IL-4 in rodent models, including studies of GBM, revealed that the release of IL-4 at the tumor site induced significant eosinophil influx, tumor rejection, and the prolonged survival of nude mice [105-107]. Another *in vivo *murine study demonstrated that IL-4-mediated tumor suppression involved the production of the cytokine interferon-gamma (IFN-γ) [108], which supports subsequent findings indicating that IL-4-transfected tumor cell vaccines promoted Th1 immunity [109]. In asthmatic patients, production of IFN-γ by CD8+ T cells has been identified [110,111]. In ovalbumin sensitized rats, production of IFN-γ by CD8+ T cells suppressed eosinophilia, and in human eosinophils, IFN-γ has also been found to enhance cytokine- (GM-CSF, IL-5) induced degranulation and superoxide anion production [112,113]. Thus, in certain cases, effective GBM tumor eradication may occur in response to IL-4 and the concomitant recruitment of CD8+ T cells and eosinophils whereby the CD8+ T cells identify specific antigens and produce IFN-γ that enhances eosinophil activation and the release of cytotoxic granules. Modifications of these events via the aforesaid SNPs (IL-4Rα, IL-13) may allow for immune evasion and tumor formation.

### Eosinophils and GBM initiation

Previous studies linking eosinophil function and tumor biology have indicated that eosinophil production of eosinophil peroxidase (EPO) and reactive oxygen species (ROS) may amplify oxidative damage and tumorigenesis in the lung [114], possibly via induced activation by the cytokine, GM-CSF, which has been shown *in vitro *to elicit these responses in human eosinophils [112]. Of note, human astrocytes and GBM tumor cells are also known to produce GM-CSF [115-117], which may enhance oxidative stress in a microenvironment involving eosinophils. Oxidative stress not only functions to induce DNA mutations but may also affect cell senescence and apoptosis in developing tumors [118]. Activated eosinophils are also known to produce eosinophil derived neurotoxin (EDN, RNase 2) and eosinophil cationic protein (ECP, RNase 3) [119]. EDN and ECP exhibit antiviral functions [120,121] that may play role in preventing HCMV induced tumor formation. EDN has also been identified as a toll-like receptor-2 (TLR2) ligand that can promote the *in vivo *activation of murine dendritic cells (DCs) [122]. In experimental GBM models, TLR2-ligands have been indicated to induce an influx of tumor-infiltrating immune cells (DCs, CD8+ T effector cells) and significant tumor regression [123,124], which raises the possibility that EDN may operate comparatively. In addition, ECP can alter cell membrane permeability and induce toxicity in cancer cell lines [121]. EDN and ECP may therefore be effectual eosinophilic components in preventing tumor formation (see Figure 2).

## Promotion

### Eosinophils and growth factors in GBM promotion

**Figure 3:**
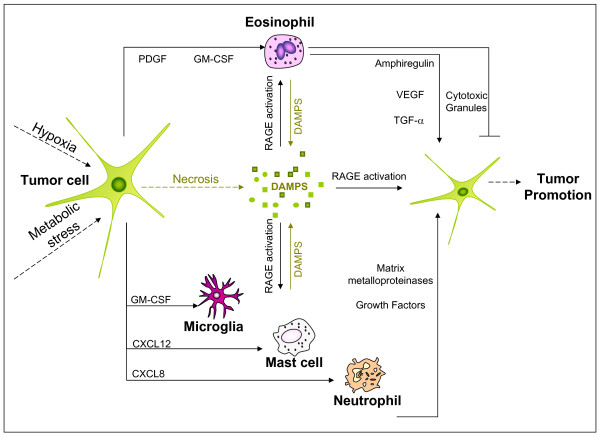
**Eosinophils in tumor promotion**. A microenvironment involving rapidly dividing cells induces tumor necrosis and the production of damage associated molecular patterns (DAMPs) which include the RAGE ligands (e.g.: HMGB1 and S100 proteins). Eosinophils and additional innate immune cells (microglia, mast cells, neutrophils) are activated by GBM mediators (GM-CSF, PDGF, CXCL12, CXCL8) and DAMPs which may in turn induce the production of growth factors (amphiregullin, TGF-α, VEGF) and matrix metalloproteinases in promoting tumorigenesis. Alternatively, in certain cases, eosinophil release of cytotoxic granules (EDN, ECP, calprotectin) may function to prevent tumor promotion.

The promotion phase of carcinogenesis involves mitogenesis that is dependent upon apoptotic inhibition and growth stimulation [125]. In GBM, the cell signal cascades that regulate the activation of members of the NF-κB transcription factor family are altered, which in turn leads to enhanced expression of the anti-apoptotic molecules Bcl-2 and survivin [126,127]. This pathway is known to be stimulated by various growth factors, ROS, and viruses such as HCMV [128]. Platelet-derived growth factor (PDGF) and PDGF receptors are expressed in GBM tumor cells and found to regulate NF-κB activation and cell proliferation [129-131]. Eosinophils likewise express PDGF receptors and PDGF has been reported to activate eosinophils [132]. Activated eosinophils may then release their cytotoxic granules and encourage anti-tumor and/or anti-viral responses during tumor promotion. Alternatively, activated eosinophils may enhance the promotion process through the production of tumor promoting growth factors (see Figure 3) [117].

Eosinophils and additional innate immune cells (microglia, mast cells, neutrophils) are activated by GBM mediators (GM-CSF, PDGF, CXCL12, CXCL8) and damage associated molecular patterns (DAMPs: e.g.: S100 proteins, high mobility group box 1) which may in turn induce the production of growth factors and matrix metalloproteinases in promoting tumorigenesis [83,84,117,132-141]. Human eosinophils stimulated *in vitro *with GM-CSF produce amphiregulin and transforming growth factor-alpha (TGF-α), which are ligands known to activate epidermal growth factor receptors (EGFR) [134,135]. In primary GBM, amplification of the EGFR gene and subsequent over-expression of EGFR protein is the most common genetic alteration [142]. Increased expression of epidermal growth factor receptors, ligands and cell signals are highly implicated in the promotion of many tumors, including GBM [143]. Of interest, GBM cell lines cultured in eosinophil-conditioned media, generated in the presence or absence of GM-CSF, demonstrated increased cell growth compared to controls [117]. Thus, because GBM tumors are known to produce GM-CSF [115-117], a paracrine loop may develop where eosinophils promote GBM development by producing amphiregulin, TGF-α, or other growth factors in response to GBM-derived GM-CSF.

### Eosinophils and RAGE in GBM promotion

Chronic inflammation associated with tumor promotion has also been linked to the activity of the receptor for advanced glycation end-products (RAGE) and RAGE ligands via an *in vitro *analysis of GBM cell lines and in an *in vivo *murine model of skin carcinogenesis [144,145]. Eosinophils and GBM tumor cells each express RAGE and RAGE ligands where cell viability and migration are reported RAGE-mediated responses in these cells [83,145]. The S100A8 and S100A9 proteins are RAGE ligands [146], DAMPs [147], markers of myeloid-derived suppressor cells [148], GM-CSF induced cytokines in eosinophils [83], and tumor promoting factors in experimental models [144,149]. Interestingly, the S100A8 and S100A9 complex, calprotectin, is indicated to induce apoptosis in cancer cell lines [150]. Benign lesions associated with normal brain aging (corpora amylacea) also express both S100A8 and S100A9 [151]. In human GBM primary tumor parenchyma, S100A8 and S100A9 have been identified with higher levels of S100A9 noted in the tumor regrowth parenchyma of patients that received primary resection plus irradiation compared to primary resection alone [152]. Of interest, radiation treatment of pelvic cancers has been found to induce eosinophilia and increase ECP serum levels [153,154]. Eosinophils cultured in GBM-cell line conditioned media *in vitro *have also been shown to release S100A9 [117]. Whether eosinophils are functional in promoting or preventing benign or cancerous lesions in the brain via S100 proteins and RAGE-mediated responses in these various disease states is not clear but the interactions may represent an important link between eosinophils and glioblastoma biology (see Figure 3).

## Progression

### GBM is a progressed disease

The progression stage in cancer biology arises from additional genetic and epigenetic events that confer phenotypic changes that are necessary for tumor cell autonomous growth, invasiveness, and migration [155]. At diagnosis, primary GBM (World Health Organization (WHO) Grade IV astrocytoma) presents at a progressed stage and is distinguished histopathologically from anaplastic astrocytoma (WHO Grade III astrocytoma) by the presence of necrosis, microvascular hyperplasia and possibly thrombosis [77]. Necrotic cell death in a developing tumor may occur in response to the increased metabolic demands of rapidly dividing cells, resulting in hypoxia, intravascular occlusion and thrombosis followed by the production of pro-angiogenic and pro-inflammatory mediators (see Figure 2) [77,88,156].

### Eosinophils and dexamethasone in GBM

Recruitment of murine eosinophils to the tumor microenvironment is indicated to occur in response to necrotic cell death [76]. In rodent models, eosinophil numbers and recruitment to the lung are reduced by dexamethasone [157,158], a common corticosteroid administered to GBM patients with peritumoral edema [159]. In some GBM cases, dexamethasone therapy (16 mg/day) has been observed to reduce the imaging of lesions on contrast-enhanced scans [160,161]. A proposed mechanism underlying this phenomenon involves a reduction in capillary permeability at the brain-tumor barrier [162]. *In vitro*, dexamethasone has been found to inhibit the release of GM-CSF from human primary T cells [163] and GBM cell lines [117]. This steroid is also indicated to reduce GM-CSF-induced survival of human primary eosinophils in tissue culture experiments [164,165]. Accordingly, the short-term response to dexamethasone therapy in a subset of GBM patients may partly reflect an increase in eosinophil death and the release of eosinophilic cytotoxic products in response to reduced tumor cell-derived GM-CSF. In addition, dexamethasone is also indicated to reduce the release of neurotrophins from human eosinophils *in vitro *[166]. Neurotrophin receptors have been identified in human GBM and are reportedly integral to disease progression [167,168]. Human eosinophils activated with GM-CSF in tissue culture have also been shown to produce vascular endothelial growth factor (VEGF), a pro-angiogenic mediator [133]. Thus, dexamethasone-induced occlusion of capilliaries may lower the recruitment of eosinophils and the production of eosinophil-derived VEGF, implicating eosinophils as potential participants in disease progression.

### Eosinophils, thrombosis, and neurotoxicity in GBM

The increased presence of eosinophils in the peripheral blood has been characterized as a pro-thrombotic condition and a potential premonitory sign of occult cancer [169-171]. Expression of tissue factor (TF) by immune cells or cancer cells is suspected to enhance the activation of the extrinsic blood coagulation pathway, resulting in the hypercoagulable state of advanced malignancy [172]. Human eosinophils *in vitro *are known to express cell membrane TF upon activation with GM-CSF [173]. Also, cell culture experiments have revealed that platelets are activated by incubation with two of the four eosinophil granule proteins (EPO, MBP), leading to the release of serotonin and β-thromboglobulin [174]. Of interest, EDN and ECP, the remaining granule proteins, have been found to induce the Gordon phenomenon, a neurotoxic event involving purkinje cell degeneration after intracerebral injection of human eosinophils into animals [175,176]. These functions of eosinophils may aid in our understanding of GBM progression and the clinical observations (motor weakness/loss, lack of coordination, altered mental function [177]) associated with the disease (see Figure 2).

## The immune response

**Figure 4:**
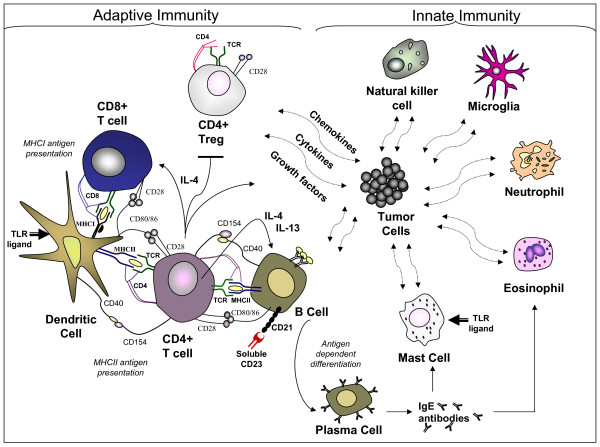
**GBM innate and adaptive immunity**. Cytokines and chemokines produced by tumor cells are indicated to alter the tumor suppressive functions of innate immune cells (natural killer cell, microglia, neutrophil, mast cell) thereby creating a microenvironment that is conducive to tumor development. Immune activators associated with allergy/asthma (IL-4, IL-13, CD23:CD21 ligation, IgE, TLR ligands) induce the recruitment and activation of immune cells (mast cells, eosinophils, natural killer cells, CD8+ T cells), the suppression of CD4+ Treg development, tumor rejection, and enhanced host survival (see text for additional details).

The innate immune response is known as the first line of defense against tumors [178]. In GBM, this includes natural killer (NK) cells, microglia, granulocytes (e.g.: eosinophils, mast cells, neutrophils), the complement system, and various immune activators (e.g.: pathogen- or damage- associated molecular patterns (PAMPs or DAMPs) or the recognition of non-self/foreign peptides) [178,179]. The adaptive immune response involves the specific identification and elimination of tumor antigens via the activation of CD8+ T cells and the generation of antibodies that target tumor-specific antigens [179,180]. The combined innate and adaptive immune responses that effectively suppress tumor formation have been termed immunosurveillance [181]. Emerging information indicating that immune cells may not only be involved in tumor prevention but also tumor development has resulted in an additional term called immunoediting [181,182]. This latter concept appears relevant to GBM in that various reports, particularly with respect to innate immune cells, indicate that the function of immune cells is altered by tumor cells to support rather than prevent tumorigenesis (see Figure 3) [117,136,137,139,183,184]. Because atopic diseases reportedly have an inverse association with glioma risk [15,18,19,185], immune activators in atopy may exhibit anti-tumor responses in GBM (see Figure 4). The cytokines, IL-4 and IL-13, are known to be up-regulated in allergy/asthma and have been characterized as integral proteins in GBM biology [95,96,98,99]. These cytokines in association with CD23:CD21 ligation, drive the generation of IgE antibodies [26]. High serum CD23 and IgE levels are associated with increased GBM patient survival and the activation of mast cells and eosinophils [28-30]. TLR-ligands are components of the innate immune system, regulators of immune activation in allergy/asthma, and recently examined adjuvants in a GBM clinical trial involving dendritic cells where improved survival in certain patient subsets was identified [178,179,186,187]. *In vitro *and *in vivo *experiments indicate that TLR-ligand activated dendritic cells or mast cells encourage CD8+ T cell recruitment [186,188,189]. Of interest, *in vivo *tumor models involving IL-4 also exhibited Th1 cell immunity as well as significant eosinophil influx, tumor rejection/suppression, and prolonged survival of the host [105-109]. Additional *in vivo *research revealed that IL-4-producing Th2 cells were critical for natural killer cell activation (perforin, granzyme-B) and tumor rejection [190]. These data concur with *in vitro *evidence indicating a function of IL-4 in suppressing the induction of tumor growth factor (TGF)-β-induced T regulatory cells [191-193]. Thus, the immune parameters in atopic disease (e.g.: IgE, TLR ligands, IL-4) may propel innate and adaptive immune responses toward tumor eradication.

## Conclusions

The mechanisms and immunobiology of GBM tumor development are not clearly known and represent areas of active investigation. In this regard, emerging evidence reveals that eosinophils may hold a functional role in the initiation, promotion and progression of developing GBM tumors. Understanding the complex nature of the innate and adaptive immune responses may foster more effective immunotherapeutic approaches in treating GBM. Because of the multiple associations of eosinophils in tumorigenesis, further study of this diverse immune cell with respect to cancer appears warranted.

## List of abbreviations

APCs: antigen presentation cells; DAMP: damage associated molecular pattern; ECP: eosinophil cationic protein; EDN: eosinophil derived neurotoxin; EGFR: epidermal growth factor receptor; EPO: eosinophil peroxidase; GBM: glioblastoma; GM-CSF: granulocyte macrophage colony-stimulating factor; HCMV: human cytomegalovirus; MHC: major histocompatibility complex; MBP: major basic protein; PAMPs: pathogen associated molecular pattern; PDGF: platelet derived growth factor; ROS: reactive oxygen species; TCR: T cell receptor; TF: tissue factor; TGF: tumor growth factor; TLR: toll-like receptor; VEGF: vascular endothelial growth factor; RAGE: receptor for advanced glycation end-products

## Competing interests

The authors declare that they have no competing interests.

## Authors' contributions

This article was written principally by CSC, with conceptual and editorial contributions from PJB. Both authors have read and approved the final version of the manuscript.
